# High-throughput 3-dimensional culture of epithelial ovarian cancer cells as preclinical model of disease

**DOI:** 10.18632/oncotarget.25098

**Published:** 2018-04-24

**Authors:** Victoria Heredia-Soto, Andrés Redondo, Alberto Berjón, María Miguel-Martín, Esther Díaz, Roberto Crespo, Alicia Hernández, Laura Yébenes, Alejandro Gallego, Jaime Feliu, David Hardisson, Marta Mendiola

**Affiliations:** ^1^ Molecular Pathology and Therapeutic Targets Research Lab, Instituto de Investigación del Hospital Universitario La Paz, IdiPAZ, Hospital Universitario La Paz, HULP, Madrid 28046, Spain; ^2^ Centro de Investigación Biomédica en Red de Cáncer, CIBERONC. Instituto de Salud Carlos III, Madrid 28029, Spain; ^3^ Department of Medical Oncology, Hospital Universitario La Paz, HULP, Madrid 28046, Spain; ^4^ Translational Oncology Research Lab, IdiPAZ, Hospital Universitario La Paz, HULP, Madrid 28046, Spain; ^5^ School of Medicine, Universidad Autónoma de Madrid, UAM, Madrid 28029, Spain; ^6^ Department of Pathology, Hospital Universitario La Paz, HULP, Madrid 28046, Spain; ^7^ Department of Gynecology and Obstetrics, Hospital Universitario La Paz, HULP, Madrid 28046, Spain; ^8^ Cátedra UAM-AMGEN, Universidad Autónoma de Madrid, Campus de Cantoblanco, Madrid 28049, Spain; ^9^ Molecular Pathology Section, Instituto de Genética Molecular y Médica, INGEMM, Hospital Universitario La Paz, HULP, Madrid 28046, Spain

**Keywords:** ovarian cancer, epithelial mesenchymal transition, 3D cell culture model

## Abstract

**Background:**

Recent reports have identified distinct genomic patterns in ovarian carcinoma, including proliferative and mesenchymal-like groups, with worse outcome. The exact mechanisms driving the onset and progression of these tumors are still poorly understood. Additionally, researchers are concerned about the correct subtype stratification of the available cell line models, and the exploration of alternatives to monolayer culture. Identification of biomarkers to stratify cell lines, characterization of important processes as epithelial-mesenchymal transition (EMT), and the use of three-dimensional (3D) cultures as alternative models could be useful for cell line classification.

**Methods and Results:**

In this work, we present a descriptive analysis of 16 commonly used ovarian cancer cell lines. We have studied their morphology in 2- and 3D culture, and their response to cisplatin, observing in the majority of them an increased resistance in 3D. We have also performed an immunohistochemical analysis for proliferation marker Ki-67, and EMT related markers to establish phenotypes. Epithelial cells tend to show higher proliferative rates, and mesenchymal cells show an increase in EMT related markers, especially when cultured in 3D conditions.

**Conclusions:**

We have stated the complex heterogeneity of ovarian cancer models, resembling primary tumors, agreeing with the argument that the cell line model for *in vitro* experiments must be carefully chosen. Our results also support that tridimensional culture could be a very helpful alternative in ovarian cancer research. Regarding EMT, a very important process for the development of this disease, some related biomarkers might be further characterized for their role in this disease development.

## INTRODUCTION

The vast majority of malignant ovarian cancer cases are carcinomas, presumably originating from the Müllerian epithelium of the ovarian surface and the fallopian tube. The World Health Organization (WHO) classification distinguishes serous, mucinous, endometrioid and clear cell as the main histological subtypes. In the serous category, accounting for over 70% of all ovarian carcinomas (OC), the existence of two different diseases is now well established, low- and high-grade tumors, with distinct morphological and molecular characteristics [1]. The first step for dissemination involves detachment from the primary tumor and shedding to the abdominal cavity as individual cells or spheroids, both usually present in patients´ ascites [2, 3]. After this initial settlement on the peritoneal mesothelial lining, cells may activate mechanisms to start the metastatic outgrowth.

One of these mechanisms, epithelial to mesenchymal transition (EMT), is a developmental program that transiently disrupts cell-cell adhesion and converts epithelial cells into more migratory and invasive mesenchymal cells [4]. This process and its regulators have also been related to chemoresistance, emphasizing its relevance with respect to recurrence of disease [5, 6]. Noteworthy, different studies have pointed proliferative and mesenchymal-like groups, based on molecular features, and related them with a worse prognosis [7, 8].

The study of relevant pathways for cancer development has been facilitated by cell-based models. Immortalized OC cell lines have been used as an alternative to freshly isolated tumor cells since they have extended lifespan and their clonal nature reduces inter-experimental variability. However, when cultured as a two-dimensional (2D) monolayer, these cell lines demonstrate low functionality and an altered phenotype compared to primary cultures. There is an urgent need to characterize the existing models in order to use the most suitable ones for research. Additionally, alternatives to the classical monolayer culture such as three-dimensional (3D) cell culture that better represent tumor features, should be considered.

The main objective of this study was to establish 3D OC models, comparing them with bidimensional culture, and to characterize them for their growth, response to treatment, and expression of EMT markers.

## RESULTS

### Optimization and characterization of two-dimension and spheroid growth OC cell lines

**Figure 1:**
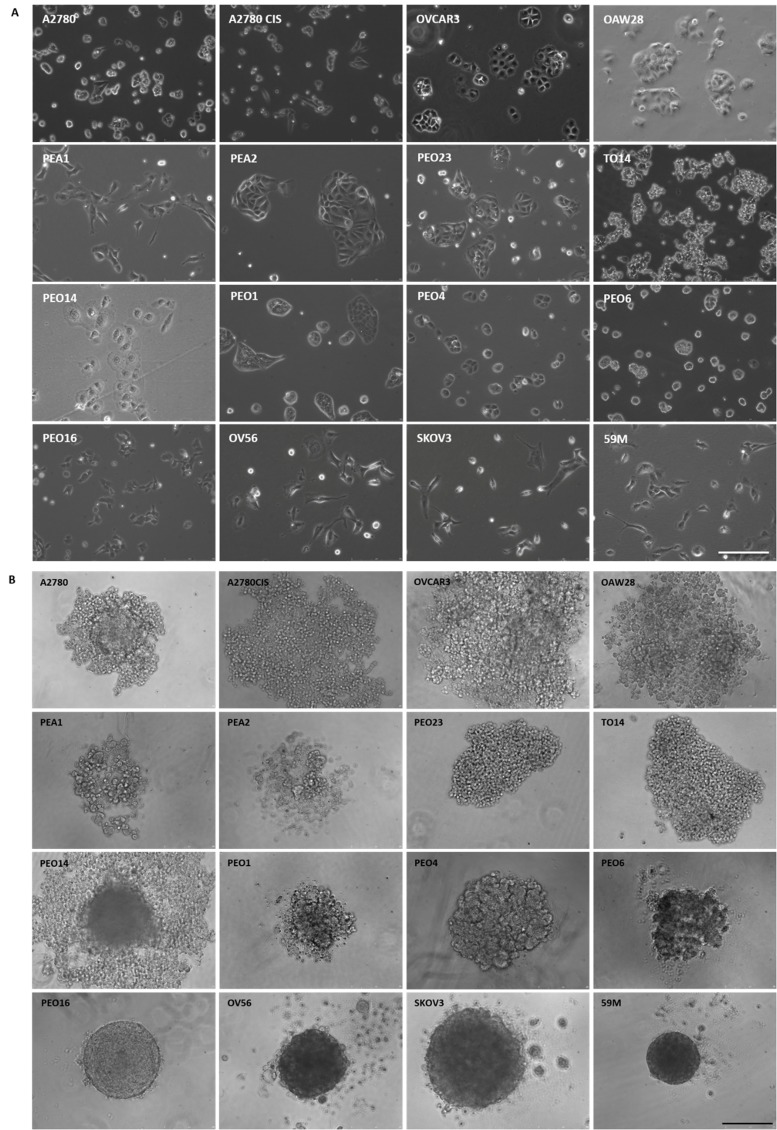
Morphological features of OC cell lines Phase contrast images. (**A**) 2D-cultures show epithelial and fibroblast-like phenotypes; (**B**) 3D-cultured cells after 4 days formed 3 different structures: loose (top 2 rows) or compact (third row) aggregates and tight spheroids (bottom row). Scale bars = 250 μm.

Two-dimensional morphology was analyzed by phase contrast imaging. We found two different patterns: round or epithelial (A2780, A2780CIS, OVCAR3, OAW28, PEA2, PEO23, TO14, PEO14, PEO1, PEO4 and PEO6), and mesenchymal or fibroblast-like (PEA1, PEO16, OV56, SKOV3 and 59M) (Figure 1A). The doubling time ranges from 24 to 58 hours (Table 1), similar to previously reported data for ovarian cancer cell lines [9]. Mean values for epithelial and mesenchymal morphology cell lines are not statistically different (36.56 ± 10.26 vs 36.21 ± 8.67, respectively).

**Table 1:** Main characteristics of ovarian cancer cell lines

Cell Line	Origin	Morphology	Spheroid type	D.T. 72 h (h)	IC_50_ ± S.D. (μM)	Resist.	IC_50_ 3D ± S.D. (μM)
A2780	T	Rounded	LA	30.72	3.51 ± 0.69	PR	NR
A2780CIS	T	Rounded	LA	31.38	13.84 ± 0.93	VR	0.66 ± 0.10
OVCAR3	A	Rounded	LA	27.22	5.01 ± 0.28	PR	44.57 ± 0.71
PEO1	A	Rounded	CA	47.83	5.52 ± 0.42	PR	NR
PEO4	A	Rounded	CA	33.34	16.11 ± 1.18	VR	NR
PEO6	A	Rounded	CA	34.27	11.94 ± 2.74	VR	NR
PEA2	A	Rounded	LA	44.13	20.80 ± 0.54	VR	NR
PEO14	A	Rounded	CA	41.28	2.68 ± 0.11	S	NR
PEO23	A	Rounded	LA	29.32	10.37 ± 0.30	R	NR
TO14	T	Rounded	LA	24.21	10.43 ± 0.55	R	NR
OAW28	A	Rounded	LA	58.45	4.59 ± 0.61	PR	NR
PEA1	A	Spindle	LA	42.06	15.47 ± 2.31	VR	NR
PEO16	A	Spindle	CS	33.76	5.48 ± 0.06	PR	7.21 ± 2.06
OV56	A	Spindle	CS	24.46	1.51 ± 0.22	S	9.34 ± 3.66
SKOV3	A	Spindle	CS	33.77	11.50 ± 1.47	R	46.03 ± 9.50
59M	A	Spindle	CS	47	9.47 ± 0.78	R	11.83 ± 0.55

Origin: T = Tumor, A = Ascites; Spheroid morphology: LA = loose aggregate, CA = compact aggregate, CS = compact spheroid; D.T. 72 h = doubling time at 72 h; S.D. = Standard Deviation; Resistance grade: S = sensitive, PR = partially resistant, R = resistant, VR = very resistant; NR = not reached.

Spheroids individually created per well on ultra low attachment (ULA) plates (Corning) were quite reproducible, as previously described [10]. Using this method, we were able to detect three different patterns for 3D growth of the cells. Some cell lines adopted a loose aggregate conformation (A2780, A2780CIS, OVCAR3, OAW28, PEA1, PEA2, PEO23, TO14), others had a more compact aggregate structure with an irregular, non-spherical, and a less defined outer perimeter (PEO14, PEO1, PEO4 and PEO6), and a third group of cells, that adopted a tight spheroid structure, with very well defined perimeters under 3D culture (PEO16, OV56, SKOV3 and 59M). Interestingly, the four cell lines that form compact spheroids share a mesenchymal or fibroblast-like morphology (Figure 1B).

When cultured in monolayer, OC cell lines do not display any morphological features to indicate histology. However, on 3D, H&E stain revealed some features, including the presence of papillae in some of them, and existence of acellular central regions due to apoptosis or necrosis, as in tumor tissue (data not shown). Changes in size, measured in bright field microscopy, could be a surrogate marker of cell growth, but only in compact spheroids, so we used Ki-67 staining for evaluation of proliferation, that diminishes with time in compact spheres such as SKOV3, and remains constant in aggregates, as represented by PEO1 (Supplementary Figure 1).

### Cisplatin (CDDP) treatment

Monolayer cultures and spheroids were treated with increasing concentrations of CDDP for 72 h. Dose-dependent reduction in cell viability was observed, but differences in drug response in 2D experiments are independent of histotype, phenotype or spheroid formation categories. Table 1 lists the IC_50_ values calculated for CDDP in ovarian cancer cell lines spheroids and monolayer cultures. Four groups of cell lines were established according to their IC_50_ values, taking IOSE cells as a reference (IC_50_ = 2 μM): sensible cells (IC_50_ < 3 μM), partially sensitive cells (IC_50_ between 3 and 6 μM), resistant cells (IC_50_ between 6 and 12 μM), and very resistant cells (IC_50_ > 12 μM).

**Figure 2:**
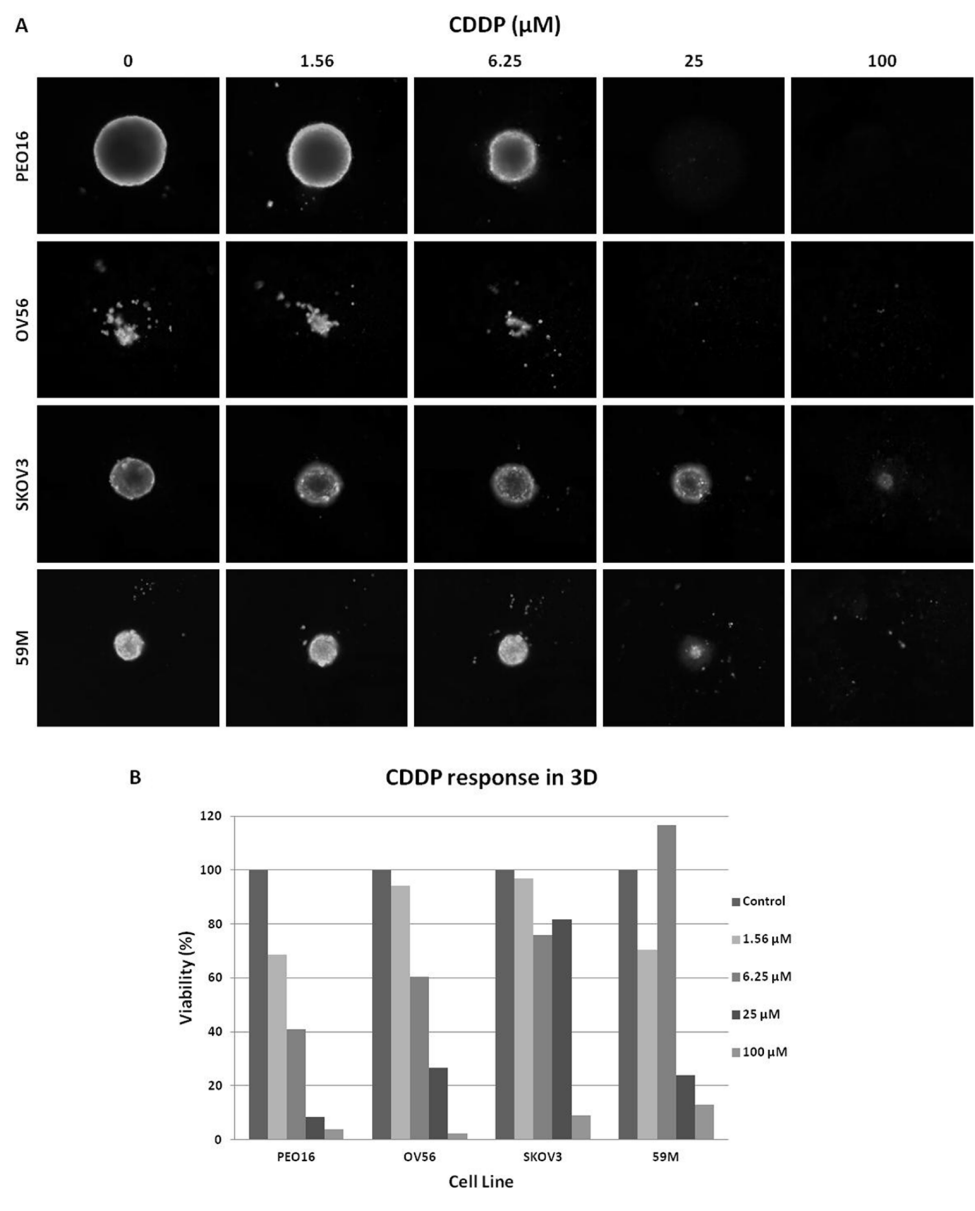
Dose-dependent sensitivity of Spheroids from 4 of the cell lines analyzed, with compact morphology (**A**) CAM stained spheroids. Cells were treated with different CDDP concentrations. Images obtained with Celigo S cytometer; (**B**) Quantification of cell viability of the cells at different CDDP concentrations calculated with mean integrated CAM intensity values.

In all cell lines, independent of their 3D growth pattern, platinum response was evaluated by a luminescent assay (CellTiter-Glo) (Table 1). Additionally, CDDP sensitivity was evaluated by size and CAM staining in the four cell lines that are able to form compact spheroids (PEO16, OV56, SKOV3 and 59M) obtaining comparable results (Figure 2). In this latter group, two cell lines maintain similar IC_50_ values (fold change <2, PEO16 and 59M) whilst the other two show an increase when cultured in 3D (6.2 and 4.0 fold for OV56 and SKOV3, respectively). The remaining group of aggregate forming cell lines, with the exception of two of them (A2780CIS and OVCAR3), did not reach the IC_50_ values when cultured on 3D conditions at the same concentration range set for 2D experiments (100 µM top concentration), and only one (A2780CIS), experimented a decrease in IC_50_ (Table 1).

### Immunohistochemical analysis

Ki-67 was employed to evaluate cell proliferation of cultures (Supplementary Figure 1 and Table 1). The percentage of positive cells is similar comparing 2- and 3D globally (42.50 ± 26.39 vs 47.37 ± 6.70, respectively); no statistical differences were observed between 3D growing patterns, although the less proliferative seemed to be the compact aggregate type (52.5 ± 18.08 for loose aggregates, 18.75 ± 5.15 for compact aggregates, and 59.12 ± 20.24 for compact spheroids). Mean values became statistically different when we clustered the cells by morphological groups, increasing on 3D for the rounded cells (36.36 ± 28.64 vs 61.00 ± 36.49, *p* < 0.05), and decreasing for spindle subtype (56.00 ± 15.16 vs 17.40 ± 9.24, *p* < 0.05).

Supplementary Table 1 includes detailed information regarding reported molecular alterations and putative histotype of the cell lines included in this study. We have found some discrepancies regarding P53 expression. When it is completely absent or very highly expressed, it is widely used as a surrogate marker for *TP53* mutation, a ubiquitous molecular sign in high-grade serous ovarian carcinoma (HGSOC) [11]. Twelve of the cell lines showed abnormal expression of P53. The remaining cell lines, OVCAR3, OAW28, PEO14 and PEO23, presenting a normal expression pattern of the protein in our study, have been reported as *TP53* mutated by different authors. PEO16, A2780 and 2780CIS showed an abnormal staining pattern; however, they are not reported as mutated on previous studies.

Table 2 summarizes the results of the inmunohistochemistry (IHC) study on EMT-related markers. For the phenotypical classification of cell lines, we used the epithelial markers E-Cadherin (ECAD) and Pan-Cytokeratin (PANCK), and the mesenchymal markers N-Cadherin (NCAD) and Vimentin (VIM). Expression of these 4 markers was not mutually exclusive in the panel analyzed. With this algorithm, we classified four cell lines as purely epithelial (PEO4, PEO6, OAW28 y OVCAR3), and five as purely mesenchymal (PEO23, TO14, PEO16, A2780 y A2780CIS); the remaining cell lines were classified as intermediate. This phenotypical classification correlates with the morphology exhibited by the cells when cultured in monolayer in 12 of the lines; the remaining cell lines (PEO23, TO14, A2780 and A2780CIS) exhibited a rounded morphology but expressed a mesenchymal immunohistotype. Interestingly, none of the compact aggregates expressed NCAD, and none of the cells forming compact spheroids expressed ECAD, and the latter remain in the mesenchymal category by this IHC classification (Supplementary Figure 2). Pooling pure and intermediate subtypes for statistical analysis, mesenchymal cells had a significant reduction on their doubling time compared to the epithelial group (41.07 ± 9.68 vs 31.83 ± 7.15, *p* = 0.04); no significant differences were observed on Ki-67 expression between 2- or 3D culture (data not shown).

**Table 2:** IHC of EMT biomarkers expression in cell lines, both in monolayer (2D) and 3D culture

Cell Line	EMT Markers												Phenotype Classification									
	SNAIL		SLUG		ZEB1		ZEB2		TWIST1		TWIST2		ECAD		PANCK		NCAD		VIM		Phenotype	
	2D	3D	2D	3D	2D	3D	2D	3D	2D	3D	2D	3D	2D	3D	2D	3D	2D	3D	2D	3D	2D	3D
**PEO4**	–	–	–	–	–	–	+	–	+	++	+	+	R	C	C	C	A	A	A	A	E	E
**PEO6**	–	+	–	+	–	–	–	–	+	++	–	+	R	R	C	C	A	A	A	A	E	E
**OAW28**	+	+	–	–	–	–	–	–	–	++	+	+	R	R	C	C	A	A	A	A	E	E
**OVCAR3**	+	+	–	–	–	–	–	–	+	++	+	+	R	R	C	C	A	A	A	A	E	E
**PEA2**	–	–	–	+	–	–	–	+	+	+	+	++	R	C	C	C	A	A	R	R	IE	IE
**PEO1**	–	–	–	+	–	–	–	+	+	+	+	+	R	R	C	C	A	A	R	R	IE	IE
**PEO14**	+	+	–	+	–	+	–	–	–	++	+	+	R	R	C	R	A	R	R	R	IE	I
**PEA1**	–	+	–	+	–	+	–	+	–	++	+	++	A	A	C	R	A	A	C	C	I	IM
**OV56**	+	+	–	+	–	+	+	+	–	++	+	+	A	A	R	C	R	R	C	C	IM	IM
**SKOV3**	–	+	–	+	+	+	–	–	–	+	+	+	A	A	C	R	R	R	C	C	IM	IM
**59M**	+	–	–	–	–	+	–	–	+	++	–	–	A	A	C	R	C	C	C	C	IM	IM
**PEO23**	–	+	–	+	+	+	–	+	–	++	+	++	A	A	A	A	A	A	C	C	M	M
**TO14**	–	+	–	–	+	–	–	+	–	++	+	++	A	A	A	A	A	A	C	C	M	M
**PEO16**	–	–	–	–	–	+	–	+	+	++	+	–	A	A	A	A	C	C	C	R	M	M
**A2780**	+	+	+	–	–	+	–	+	+	++	–	+	A	A	A	A	R	R	C	C	M	M
**A2780CIS**	+	+	+	–	–	+	–	+	+	++	+	+	A	A	A	A	R	R	C	C	M	M

Phenotype: E = epithelial, IE = intermediate epithelial, I = intermediate, IM = intermediate mesenchymal, M = mesenchymal; A = absent, C = conserved, R = reduced, + = positive (moderate expression), ++ = positive (high expression), – = negative.

We also assessed the expression of master regulators of the EMT process in 2- and 3D culture conditions by IHC (Table 2). From the 16 cell lines analyzed on flat culture, markers were expressed as follows: Seven (44%) are positive for SNAIL, 2 (12%) are positive for SLUG and ZEB2, 3 (19%) for ZEB1, 9 (56%) for TWIST1, and 13 (81%) for TWIST2. The expression is variable and we did not find any relation with the 3D growth patterns as aggregate or compact spheroids, response to platinum or proliferation, and the expression of the analyzed markers. Globally, there is an increased expression of all the EMT markers analyzed in 3D. Specifically, splitting cells according to the epithelial or mesenchymal IHC phenotype, and comparing 2- and 3D culture, we observed an increased expression of SLUG and TWIST markers in the epithelial group and more expression of ZEB1, ZEB2, TWIST1 and TWIST2 in the mesenchymal type, when grown under low attachment conditions.

## DISCUSSION

OC is traditionally divided into five main histological subtypes: serous (low- and high-grade), endometrioid, clear cell and mucinous. HGSOC accounts for more than 70% of cases, and is responsible for the majority of ovarian cancer related deaths [12]. Recently, large scale molecular profiles have confirmed the existence of different molecular subtypes in HGSOC (proliferative and mesenchymal-like), indicating that ovarian cancer is an heterogeneous disease, with different prognosis [7, 13].

Established cell lines are the most common model in cancer research, and their use has contributed to the understanding of cancer biology in the last decades, but they also have limitations. It is now widely accepted that some of the most frequently used ovarian cancer cell lines, including A2780, SKOV3 and OVCAR3, do not seem to be the most suitable models for the study of HGSOC [14–16].

We agree with other authors that the optimal cell line for *in vitro* studies must be carefully chosen, depending on numerous factors, including the endpoint of the study, growth characteristics, or histological or genomic background [10, 17].

2D culture is easy to handle and highly reproducible, but an objection to this model is that it does not totally mimic some properties of tumors, and sometimes fails to reproduce *in vivo* drug efficacy. More recently, 3D cell culture models, expected to bridge the gap between 2D and animal models, are becoming more widely used in scientific research, including drug screening and new compounds development fields. Ovarian cancer has common tumor growth characteristics, such as a hypoxic environment, absent in traditional culture models. However, it also has some unique features, such as peritoneal spread as individual cells or aggregates. For all these reasons, 3D unattached culture could be a more representative model for these tumors.

Previous work has been published on ovarian cancer spheroids culture using different methods [18–21]. However, these models that overcome some of the limitations of 2D, as the architectural growth of the tumor, are not yet fully attained. Reproducibility and easy management in the 2D setting are more complicated to perform on 3D.

In this study, we characterized ovarian cancer spheroids from 16 commonly used cell lines, using ULA plates, in a highly uniform way, as previously described [10]. This method emulates more precisely the growth as aggregates (loose or compact) but also as solid tumors (compact spheroid), depending on the cell line. Interestingly, in this panel all cell lines with mesenchymal morphology formed compact spheroids. The compactation level on 3D had also been previously correlated to the spindle cell morphology by other authors [22].

Regarding compact spheroids experiments, assessment of cell number and days of incubation is crucial. An estimated diameter of 400 μm could be optimal to mimic the diffusion state in the tumor, which is about 100 μm in depth for nutrients and oxygen, and 14 days as final time point was also effective to avoid excessive necrotic areas [23–25]. Proliferation of cells within the compact spheroids decreased over time but this effect is not necessarily accompanied by a decrease in the spheroid volume, which can remain tight and intact but with no viable cells. Therefore, vital staining with CAM could be used as direct indicator of cellular health, and it can also be coupled to Hoechst and PI for a more precise analysis on spheroids viability [26, 27].

Differences in cellular response to platinum were determined to be cell line specific. This can be explained because every cell line was established at a different treatment time-points: before or after first line, on different relapse states, or even after alternative chemotherapy regimes. In this study, 2- and 3D comparison in the compact spheroids group, show two cell lines that retain similar IC_50_ values, and other two that experiment an increase. The same behavior was seen for the aggregate 3D group where, with the exception of one cell line, all had an increase in the IC_50_ values. Moreover, most of them do not even reach this value under the same CDDP concentration range tested on 2D. These results are in agreement with the reported by other authors, supporting that cells tend to be more resistant to treatment when growing on 3D [28]. In advanced ovarian cancer, a high percentage of patients will develop ascites, and most of them will finally relapse, experiencing an increase in platinum resistance with recurrence. Under this scenario, and taking into account our results regarding the increased resistance observed in 3D culture, it could be worth considering unattached growth not only for compact spheroids but also for aggregates, to characterize resistance mechanisms in OC, as suggested by other authors [29]. The combination of a very reproducible approach for 3D culture, with reproducible single spheroids or aggregates per well, combined with a semi-automated quantification system as Celigo, could be a suitable model for ovarian cancer resistance research. This methodology allows the measurement of different culture features other than growth, such as migration, invasion or cell cycle progression, turning it into a very attractive approach [27, 30–32].

P53 pathway is disrupted in the majority of human cancers, mainly through mutation or deletion of *TP53* itself, and these situations have been associated with poor prognosis and chemoresistance in different types of tumors [33, 34]. In ovarian cancer, P53 is a useful marker to distinguish between HGSOC, mutated in 96% of the cases [35], and low-grade serous ovarian carcinoma (LGSOC), were mutations are very rare [1, 36]. In this study, we have detected an abnormal expression of P53 by immunohistochemical analysis in 12 out of 16 cell lines, which has been suggested to be a surrogate marker for *TP53* mutation status [37]. However, we found discordant results in other 4 cell lines regarding their reported mutations on literature. These results are in agreement with the observation that P53 IHC accuracy is not perfect, and a combination of different markers might be used in order to better classify available cell lines [15, 17, 38].

We have also evaluated Ki-67 proliferation activity, which revealed a similar proliferation rate in 2- and 3D culture, although it has been shown to decrease in other reports [39]. Interestingly, if we split the sample by morphology, rounded cells increase their proliferation on 3D meanwhile the spindle ones decrease it.

During ovarian cancer progression, both EMT and the reverse process, mesenchymal-epithelial transition (MET) occur in a dynamic way, and a related phenotype has been pointed to have a more aggressive behavior [35, 40]. We have analyzed a selected panel of EMT markers, and we have observed co-expression of epithelial and mesenchymal markers, supporting the complexity and the dynamism of the process. This plasticity has been previously reported in ovarian cancer, particularly in HGSOC, were the balance between the epithelial and mesenchymal phenotypes is complex [4]. We also observed co-expression of different EMT regulators, grouped differently depending on the phenotypic background but we were not able to discriminate CDDP response rates regarding mesenchymal or epithelial features, probably because EMT is not the only process implicated in resistance mechanisms to platinum therapy, as previously reported [41]. Another explanation could be that we have only used a discrete set of EMT regulators, supported by the fact that the Cancer Genome Atlas Research Network pointed the mesenchymal subtype as one of the groups with a worse outcome. More research in the field should be done to identify related biomarkers [35, 40].

In most cell lines, there is an increased expression of master EMT regulators in 3D models comparing with 2D. Other authors have described changes in EMT regulators between 2 and 3D ovarian cancer models and this could be in agreement with the role of the EMT process in disease dissemination, when the cells are growing unattached, like the ascitic cells in the abdominal cavity, before seeding the peritoneum [42]. Therefore, 3D culture could be a more suitable model for the study of EMT and related processes to further characterize the role of this mechanism in OC, and to identify additional biomarkers of aggressiveness.

## MATERIALS AND METHODS

### 2- and 3D culture conditions

Ovarian cancer cell lines were obtained from the European Collection of Authenticated Cell Cultures (ECACC), and cultured based on the guidelines of the repository. Cells were regularly tested for mycoplasma infection and discontinued after 15 consecutive passages. Some of these lines were established from the same patient during the course of disease, and had received different treatment schemes prior to their establishment: PEA1/PEA2, PEO1/PEO4/PEO6 and PEO14/PEO23/T014 [43]. The cells were maintained in Roswell Park Memorial Institute (RPMI) or Dulbecco’s Modified Eagle Medium (DMEM) (Lonza, Basel, Switzerland) supplemented with 10% Fetal Bovine Serum (FBS) and 100 U/ml penicillin–streptomycin (Sigma-Aldrich, MO, USA). Immortalized Ovarian Surface Epithelium (IOSE) cells were obtained from the Canadian OvCaRe Cell Bank and grown in a combination of 199 and MCDB105 (1:1) media (Sigma-Aldrich) with 5% FBS and 50 µg/ml gentamicin. All cells were incubated at 37° C in a 5% CO_2_ incubator. Spheroids were cultured using ULA plates (Corning, NY, USA) as previously described [10], for up to 14 days.

### Cell proliferation

For monolayer experiments, cells were seeded at a density of 6 × 10^3^ cells per well in 96-well plates (MW96). Cellular confluence was measured at different time points (0, 24, 48, 72 and 96 h). For 3D experiments, cells were seed at a variable density, set before for each cell line (from 500 to 10000 cells per well) in ULA plates. Growth was tracked measuring spheroid diameter at days 4, 7, 10 and 14. Image acquisition and analysis were performed with Celigo S plate cytometer (Nexcelom, MA, USA).

### CDDP effects on ovarian cancer cell lines

Monolayer culture experiments were carried out in a similar way as proliferation assays and 24 hours after seeding, cells were exposed to different concentrations of (CDDP) for 72 h. After this time, cellular confluence was measured with Celigo S and IC_50_ values were calculated using linear regression with GraphPad Prism 7 software (GraphPad Software, CA, USA).

For 3D experiments, after 4 days of culture, spheroids were exposed to CDDP for 72 h. Cell viability was measured using CellTiter-Glo Luminescent assay (Promega, WI, USA). Additionally, in compact spheroids, cells were treated with a fluorescent staining solution containing calcein-AM (CAM) (BD Biosciences) to detect live cells, propidium iodide (PI) (Sigma-Aldrich) for dead cells and Hoechst 33342 (Sigma-Aldrich) to counterstain all nuclei. Mean CAM intensity values obtained by Celigo analysis were used to calculate IC_50_ by using linear regression.

### Histological and immunohistochemical analysis

Histological studies were performed both in cell pellets from monolayer cultures and aggregates or spheroids. Cell pellets were collected, fixed with 70% ethanol for 24 h, and paraffin embedded. Spheroids were fixed in 4% paraformaldehyde solution, embedded in 4% noble agar (Sigma-Aldrich) and then paraffin embedded. Sections were stained with haematoxylin and eosin (H&E).

We performed IHC for the markers described below. Briefly, four-μm sections were cut with a semiautomatic microtome HM3508 (MICROM), deparaffinized and rehydrated in water. Antigen retrieval was performed in a DAKO PT Link (Glostrup, Denmark). Peroxidase activity was blocked with Dako Protein block for 10 minutes (containing 0.25% casein in PBS), then incubated for 30 minutes with primary antibodies, detected with Dako Envision Plus kit, and counterstained with haematoxylin. Antibodies used include: Ki-67 (#IS626), P53 (#IS616), ECAD (#IR059), PANCK (#IR053), VIM (#IR630), all from Dako; NCAD (#ab1221), Snail (#ab135708), Slug (#ab38551), Zeb1 (#ab180905), Twist (#ab50581), and Twist2 (#ab57997), all from Abcam (Cambridge, UK), and Zeb2 (#sc-48789) from Santa Cruz (TX, USA).

Evaluation of immunohistochemical stains was performed as follows: ECAD, NCAD, PANCK, and VIM membrane staining was evaluated as conserved (C), reduced (R) or absent (A); cytoplasmic immunostaining of Snail, Slug, Twist1 and Twist2, and nuclear expression of Zeb1 and Zeb2, was evaluated as positive (moderate or high, when there is possibility of discriminate categories) or negative. P53 and Ki-67 were analyzed as a percentage, for ki-67 as continuous value of positive stained nuclei, and for P53 as two categories: aberrant expression (less than 10% or more than 60% positive nuclei), and normal expression (ranging from 11% to 59%), as previously described [33, 44, 45].

Phenotypical classification of the cell lines was done according to the expression of epithelial (ECAD/PANCK) and mesenchymal (VIM/NCAD) markers. Cells were classified as purely epithelial or mesenchymal when they only expressed epithelial or mesenchymal markers; intermediate phenotypes were established when co-expression of both groups of markers was observed.

### Statistical analysis

Results are representative of at least three independent experiments in triplicate to sextuplicate, and are represented as mean ± standard deviation (SD), except 3D experiments which were performed once with 6 replicates for each condition. Chi-Square test and Fischer’s exact test (2-sided) were performed for comparisons, and these were considered significant when *p*-values < 0.05. Graphs and statistical analysis were performed using Microsoft Office Excel (Microsoft, WA, USA), SPSS version 15.0 (IBM Corporation, NY, USA), and GraphPad Prism 7 (GraphPad Software).

## CONCLUSIONS

Spheroids show different features compared with 2D culture. Particularly in OC, epithelial ovarian cancer cells growing as spheroids are frequently detected in the ascites of the patients. We have successfully used a reproducible technique to obtain uniform ovarian cancer spheroids over 16 established cell lines, and characterized their growth, proliferation, and drug response. Compelling evidence has suggested the critical role of EMT in cancer development and progression, but their regulation mechanisms need to be further characterized. We propose the use of 3D models for this purpose that, with optimal instrumentation and computational coupled resources, could represent a promising model for high throughput studies.

## SUPPLEMENTARY MATERIALS FIGURES AND TABLE


