# Supplementary material for: High-throughput 3-dimensional culture of epithelial ovarian cancer cells as preclinical model of disease
Source: Oncotarget. 2018 Apr 24;9(31):21893–903. doi: 10.18632/oncotarget.25098 (PMC5955171; doi:10.18632/oncotarget.25098)
Supplement: Supplementary file 1 [file oncotarget-09-21893-s001.pdf]

High-throughput 3-dimensional culture of epithelial ovarian cancer cells as preclinical model of disease

SUPPLEMENTARY MATERIALS

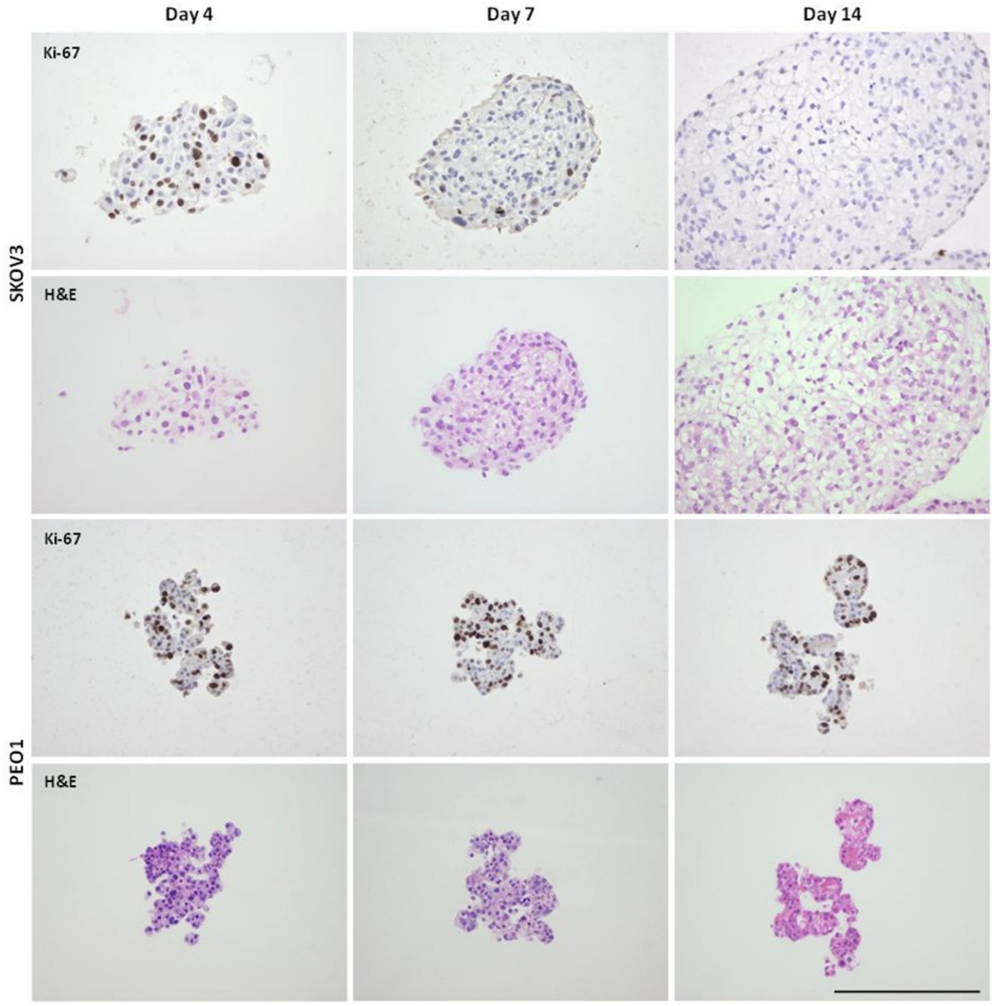

Supplementary Figure 1: Spheroid internal structure, morphology and proliferation. Spheroids were cultured on ULA plates, collected and fixed at days 4, 7 and 14, paraffin embedded, sectioned, and stained with Ki-67 and H&E. Images of spheroid mid-sections. Scale bar = 250 µm.

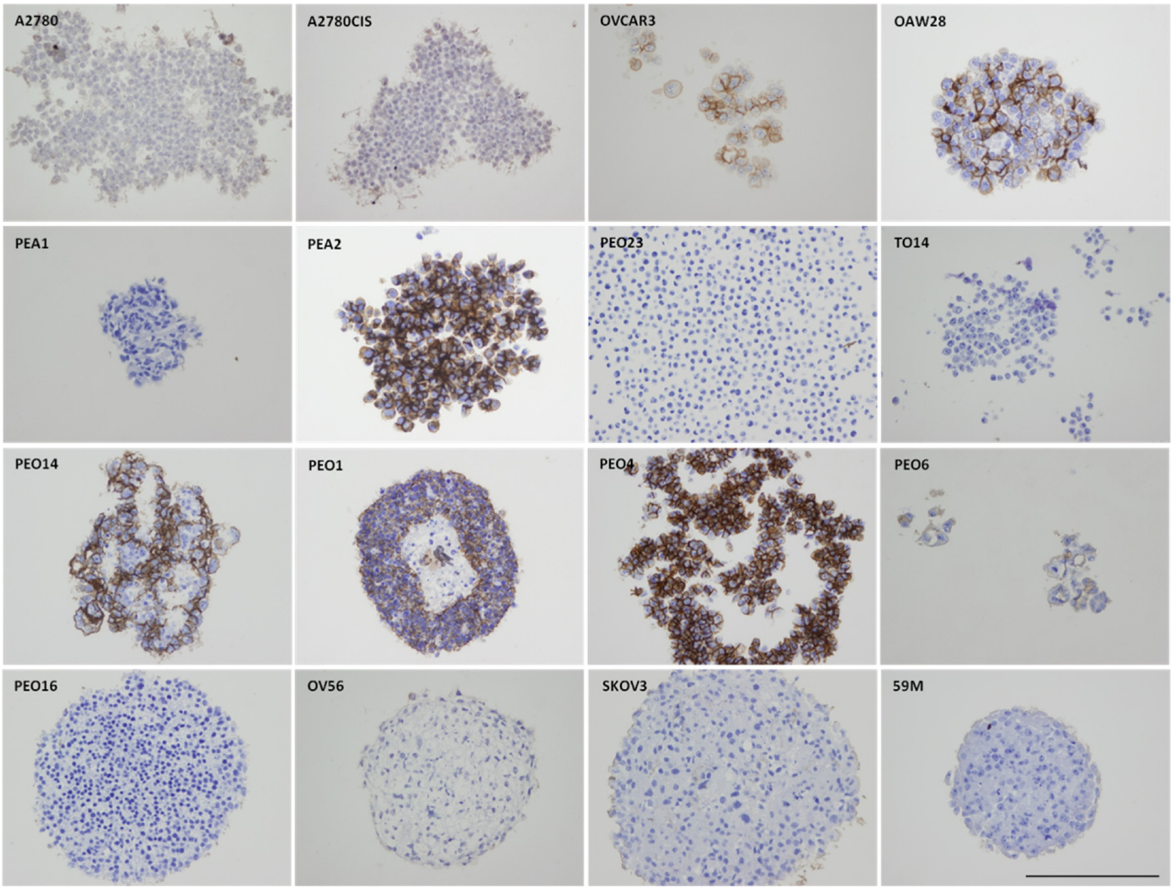

Supplementary Figure 2: ECAD expression. Spheroids collected at day 7, were paraffin embedded, sectioned and stained with specific antibodies (brown) and haematoxylin (blue) to stain the nuclei. Scale bar = 100 µm.

Supplementary Table 1: Previously described mutations in ovarian cancer cell lines, and IHC results for Ki-67 and P53

Cell Line	Described Mutations	Putative Histotype§	Ki-67		P53
			2D	3D	
A2780	BRCA2 [39], PIK3CA [15, 17, 39], BRAF [15, 17], ARID1A [1, 2, 3], PTEN [15, 17, 39], Wnt/BCAT [39], HRR [39]	EC	80	40	Abn
A2780CIS	BRCA1, PIK3CA, ARID1A, PTEN, Wnt/BCAT, HRR [39]	EC	40	95	Abn
OV56	TP53 [39], KRAS [15, 17, 39], ARID1A [15, 17, 39], PTEN [15, 17, 39], Wnt/BCAT [39], MMR [17]	EC	40	30	Abn
PEA1	TP53, Wnt/BCAT, HRR [39]	HGSOC	70	12	Abn
PEA2	TP53, HRR [39]	HGSOC	100	1	Abn
PEO1	TP53, BRCA2, HRR [39]	HGSOC	40	20	Abn
PEO4	TP53, BRCA2*, HRR [39]	HGSOC	10	25	Abn
PEO6	ND	HGSOC	10	70	Abn
PEO16	BRCA2, HRR [39]	HGSOC	40	10	Abn
PEO14	TP53, Wnt/BCAT [39]	HGSOC	20	95	N
PEO23	TP53, Wnt/BCAT [39]	HGSOC	25	95	N
TO14	ND	HGSOC	32	60	Abn
OVCAR3	TP53 [15, 17, 39], HRR [39]	HGSOC	25	95	N
59M	TP53 [15, 17]	HGSOC	70	10	Abn
OAW28	TP53 [15, 17, 39], KRAS [15]	MC	30	40	N
SKOV3	TP53 [39], PIK3CA [15, 17, 39], ARID1A [15, 17, 39], ERBB2 [15, 39], Wnt/BCAT [39], HRR [39]	Mx	60	25	Abn

(*): silent mutation, (§): According to dual carcinogenesis model [1]. HRR: Homologous Recombination Repair pathway; ND: not described; MMR: Mismatch Repair pathway. EC: Endometrioid Carcinoma; HGSOC: High-Grade Serous Ovarian Carcinoma; MC: Mucinous Carcinoma; Mx: Mixed Origin. P53, Abn: Abnormal expression and N: Normal expression by IHC.
